# Potential antioxidant and anti-inflammatory impacts of *Salvia officinalis* leaves extract on mice experimentally infected with *Trichinella spiralis*

**DOI:** 10.1007/s11259-025-10875-w

**Published:** 2025-09-30

**Authors:** Marwa Safi-eldin, Mahmoud M. Abdelfattah, Safaa Barghash, Ameen A. Ashour, Hoda A. Taha

**Affiliations:** 1 Department of Zoology, Faculty of Science, Ain Shams University, Cairo, Egypt; 2 Animals and Poultry Health Department, Desert Research Center, Cairo, Egypt

**Keywords:** *Trichinella spiralis*, *Salvia officinalis*, Experimental infection, Gene expression, Albendazole, Oxidative stress

## Abstract

Trichinellosis is a dangerous food-borne zoonotic infection with a global spread. Treatment is currently insufficient. The study aimed to assess the potential therapeutic effects of Sage (*Salvia officinalis*) leaves extract in comparison to albendazole against *Trichinella spiralis* infection in mice. Seventy mice were classified into six groups, non-infected control, non-infected receiving *Salvia officinalis* extract, infected control, infected and treated with albendazole (50 mg/kg), infected and received *Salvia officinalis* extract (250 mg/kg), and infected and treated with combination of 25 mg/kg albendazole and 250 mg/kg *S. officinalis* extract. Mice were sacrificed at 7- and 37-days post-infection for intestinal and muscle phases, respectively. Efficacy was assessed through parasitological parameters, anti-inflammatory and oxidative stress markers. Our finding revealed a significant reduction in the adult worms and larvae counts of *T. spiralis* in albendazole (97.3% (*P* < 0.01), 74.19% (*P* < 0.05), *S. officinalis* extract (80% (*P* < 0.05), 60% (*P* < 0.05) and combined treatment led to the highest reduction rate (91.9% (*P* < 0.05), 81.4% (*P* < 0.01). *S. officinalis* extract boosted the immune response by reducing NO and MDA levels while increasing SOD, CAT and GSH in intestinal and muscular tissues. Additionally, it improved the inflammatory response, Albendazole and *S. officinalis*, especially in combination, have effectively suppress inflammatory and fibrotic gene expression (FN1, TNFα, TGF-β, and IL-10) indicating synergistic therapeutic efficacy. In conclusion, *S. officinalis* extract can be considered a practical and effective alternative herbal drug in treating trichinellosis.

**Supplementary Information:**

The online version contains supplementary material available at 10.1007/s11259-025-10875-w.

## Introduction

Trichinellosis is a zoonotic disease caused by the nematode genus *Trichinella*. It affects a wide range of hosts, including horses, pigs, mice, birds, and humans (Huang et al. 2020; Salama et al. 2022). According to the World Health Organization (WHO), *Trichinella* species are globally distributed zoonotic parasites that can infect humans through the consumption of raw or undercooked meat, particularly pork or game meat. It is estimated that thousands of cases occur annually worldwide, with varying incidence depending on regional dietary habits and food safety measures. The disease can cause severe illness and, in rare cases, death, with mortality rates reported around 0.2% in some outbreaks (WHO 2020). The primary human infection is consuming undercooked pork meat contaminated by infective larvae (Maged et al. 2023). In humans, symptoms begin with gastrointestinal irritation followed by periorbital edema, swelling around the eyes, muscle pain, fever, myalgia, and pneumonia and can be fatal depending on the severity of the infection; death can occur 4–6 weeks after the infection (**՛**Pavić et al. 2020). The life cycle of *Trichinella spiralis* consists of enteral and parenteral phases. The enteral or intestinal phase clinically presents with abdominal symptoms such as diarrhea and abdominal pain. The parenteral or muscular phase presents with periorbital edema, myalgia, and muscle weakness (Abou Rayia et al. 2017; Salama et al. 2022). Long-term trichinellosis causes significant interaction with the immune system of the host, causing inflammation in the muscles that are affected (Elgendy et al. 2020).

Infection is typically treated with benzimidazole derivatives such as albendazole or mebendazole. However, both drugs have been linked to numerous side effects and have limited effectiveness against the encapsulated or newly hatched larvae of *T. spiralis*. Additionally, most of them are forbidden for infants under two and pregnant women (Yadav and Temjenmongla 2012). Therefore, studying alternative therapies is important because of their great efficacy and dependability, having less toxicity, and being free from adverse effects, as well as their affordability.

Sage (*Salvia officinalis*) is a plant in the mint family Lamiaceae; it is an evergreen plant with short woody stems, greenish-grey leaves, and blue to purplish flowers. It has long been used in medicine and recipes. Its leaves contain essential oils and tannins that can help to facilitate digestion with anticonvulsant, anti-fever, anti-diabetic, antiseptic, antibiotic, antifungal, and anti-parasitic effects (Amirmohammadi et al. 2014; Abouelsoued et al. 2020).

*Salvia* extracts also show potential against various parasitic infections, likely by disrupting parasite metabolism, inhibiting development, and modulating host immune responses (Llurba-Montesino and Schmidt 2018). Previous studies have demonstrated that *S. officinalis* has an antiparasitic effect on the parasites *Syphacia obvelata*, *Aspiculoris tetrapetra*, and *Hymenolepis nana* (Amirmohammadi et al. 2014), *Leishmania major* (Nikmehr et al. 2014), *Cryptosporidium parvum* (Abouelsoued et al. 2020; Al-Dulaimi et al. 2021), *Eimeria tenella* (Sidiropoulou et al. 2022), and *Ichthyophthirius multifiliis* (Özil 2023). Also, Alcoholic extracts of *S. officinalis* exhibit strong protoscolicidal activity against *Echinococcus granulosus* cysts in vitro (Yones et al. 2011). Moreover, diterpenes and flavonoids in sage and related *Salvia* species demonstrate antiprotozoal efficacy against protozoans, including *Trypanosoma* spp. (Llurba-Montesino and Schmidt 2018).

Moreover, *S. officinalis* has been used as a treat for sore throats, to lessen hot flashes during menopause and to improve menstrual cycle regularity, to combat gastroenteritis and other infections, to enhance liver function and lipid status, to enhance appetite and digestion, and to enhance mental capacity (Jakovljević et al. 2019). Numerous studies indicated that *S. officinalis* possesses antibacterial capabilities and radical scavenging properties (Risaliti et al. 2019; Garg and Kumar 2024), anti-inflammatory properties (Coisin et al. 2021; Garg and Kumar 2024), and anticancer effects (Manuele et al. 2021a, b; Xu et al. 2022).

Thus, the current study was conducted to evaluate the parasiticidal properties and anti-inflammatory effects of *S. officinalis* extracts against intestinal and muscular phases of *T. spiralis* infection in comparison with albendazole (reference drug) in experimentally infected mice.

## Materials and methods

### Experimental animals and parasite

A total of seventy laboratory-bred, male CD1 Albino mice, 5 weeks old, with an approximate weight of ~ 30 g, were used. All animals were housed in clean cages and given a diet and water and kept under suitable light and temperature. The experiment was carried out in the zoology department laboratories, Faculty of Science, Ain-Shams University, Egypt. The strain of *T. spiralis* was obtained from Theodore Bilharz Research Institute, Giza, Egypt. Mice were infected orally with 250 *T. spiralis* muscle larvae (ML) per mouse.

### Reference drug

Albendazole (Evazole^®^) 400 mg/10 ml was obtained from EVA Pharma, Cairo, Egypt, in oral suspension form. Each mouse received a dose of 50 mg/kg orally for 3 consecutive days from the 2nd day post infection for sub-groups, and at day 30 post infection for sub-groups B, according to Attia et al. (2015) and Salama et al. (2022).

### Plant extract

Sage leaves were collected from the Egyptian local markets. 250 g of sage leaves were cleaned and dried at room temperature and then were ground with a blender. An amount of 50 g of sage leaves powder was macerated in a dark place in 80% ethanol (400 mL) for 4 days at room temperature with shaking. The extract was filtrated with Whatman filter paper, and ethanol was evaporated in an oven at 50 °C to obtain viscous residues of crude plant extracts. The extracts were dried, weighed, and finally dissolved by 1% Tween 80 (w/v) to obtain a 10% Liquid extract. Ethanolic extracts were orally administered at a dose of 250 mg/kg body weight (Shahrzad et al. 2014; Maliki et al. 2021). Finally, phytochemical analysis was performed for the extract.

### Approximate quantitative chemical analysis of S. officinalis

Well-ground powder from *S. officinalis* leaves was subjected to quantitative phytochemical screening to assess the concentration of alkaloids (Woo et al. 1977), flavonoids (Karawya and Aboutabl 1982), saponins (Honerlogen and Tretter 1979), tannins (Van Buren and Robinson 1981), total phenols (Snell and Snell 1953), total lipids, total nitrogen, total proteins (AOAC Snell 1990), and total carbohydrates (Dubois et al. 1956).

### Experimental design (Fig. 1)

**Fig. 1:**
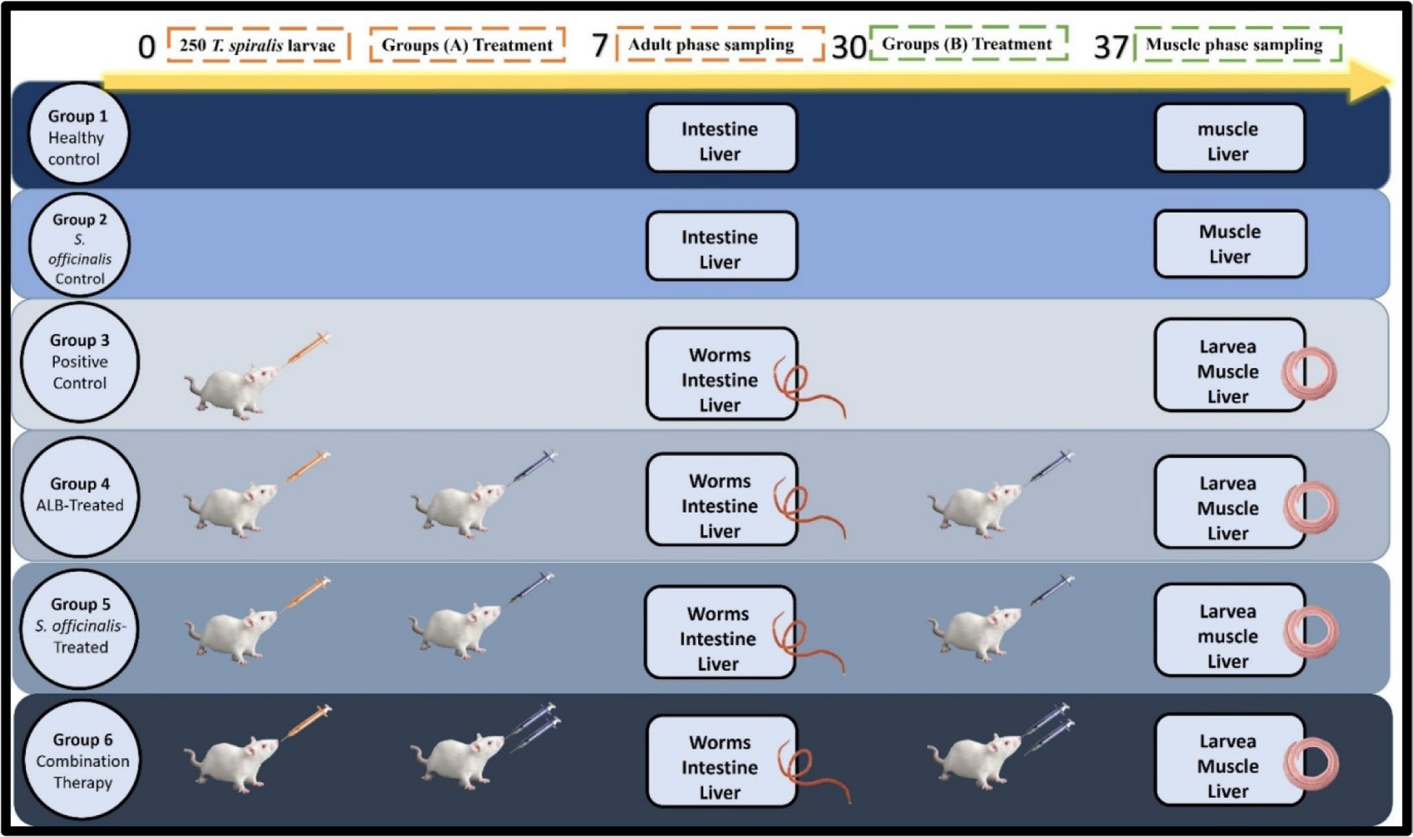
Schematic diagram of the experimental design. The figure illustrates the grouping, infection, treatment schedule, and sampling timeline used in the study

As described in Fig. 1, the animals were randomly divided into six main groups, each with two subgroups (A and B) of 10 mice, except for Group 3, which included 20 mice. Subgroup A animals received treatment starting on day 2 post-infection (p.i.) and were sacrificed on day 7 p.i., while subgroup B received treatment from day 30 p.i. and were sacrificed on day 37 p.i. The groups were as follows: **Group 1** (Negative Control): Non-infected, non-treated mice administered distilled water with Tween 80 for 5 days. **Group 2** (*S. officinalis* Control): Non-infected mice given 250 mg/kg *Salvia officinalis* extract. **Group 3** (Positive Control): Infected with 250 muscle larvae (ML) and left untreated. **Group 4** (Albendazole-Treated): Infected and treated with 50 mg/kg albendazole for 3 days. **Group 5** (*S. officinalis*-Treated): Infected and treated with 250 mg/kg *S. officinalis* extract for 5 days. **Group 6** (Combination Therapy): Infected and treated with a combination of 25 mg/kg albendazole and 250 mg/kg *S. officinalis* extract for 5 days.

### Adult worms count

On day 7, after mice anesthetization, the small intestine was removed and then washed with physiological saline; it was cut lengthwise along its entirety, separated into small pieces, and soaked in physiological saline at 37 °C for 3–4 h. The intestine was vigorously shaken in the saline, washed again, and then discarded. Adult worms were settled after 30 min, then were collected in a petri dish and counted under a dissecting microscope at X 20 power (Shin et al. 2008).

### Larval burden in muscle tissue

Approximately one gram of muscle tissue was isolated from the entire carcass of each mouse and placed in a solution of 1% pepsin and 1% HCl in distilled water. The muscle was incubated at 37 °C for 2 h while being agitated with an electromagnetic stirrer. The digested product was initially filtered to remove larger particles using a sieve, followed by collecting larvae on another sieve, then washing them three times before suspending them in 150 ml tap water in a conical flask. After letting the larvae settle, the liquid on top was discarded, and the larvae were counted under the microscope (Attia et al. 2015; Salama et al. 2022; Hamed et al. 2022).

### Histopathological examinations

Intestinal tissue (1 cm) was collected from the small intestines of mice euthanized on day 7 post-infection. Diaphragm samples were collected from mice euthanized on day 37 p.i. All samples were fixed in 10% buffered formalin for 48 h, rinsed in distilled water for 30 min, dehydrated in a graded alcohol series, cleared in xylene, and embedded in paraffin wax. Paraffin Sects. (4–5 μm) were stained with hematoxylin and eosin (H&E) and examined microscopically for histopathological changes (Bancroft et al. 1996; Shalaby et al. 2010; Suvarna et al. 2018).

Histological changes in intestinal tissues were evaluated using a semi-quantitative scoring system. Scoring parameters for intestinal tissue included immune cell infiltration, goblet cell hyperplasia, crypt hyperplasia, and villus atrophy. Each parameter was graded on a scale from 0 to 3, where 0 indicated no change, 1 mild, 2 moderate, and 3 severe alterations.

Morphometric analysis of intestinal tissue was performed to measure villus height, crypt depth, and the villus height to crypt depth ratio using ImageJ software. Villus height was measured from the tip of the villus to the villus-crypt junction, while crypt depth was measured from the base of the crypt to the villus-crypt junction. For each sample, at least 10 well-oriented and intact villi and their corresponding crypts were selected randomly, and the mean values were calculated. The ratio was then determined by dividing the average villus height by the average crypt depth, providing an indicator of mucosal integrity and regeneration.

### Determination of antioxidant enzymes and oxidative stress parameters

#### Tissue homogenate

From all groups, 0.1 g Liver tissue samples were taken from each mouse and homogenized in 900 µl ice-cold phosphate-buffered saline to make 10% (w/v) homogenate and then centrifuged at 4 °C and 10,000 rpm for 20 min; the supernatant was kept at −80 °C until used. The homogenate was used for determination of total proteins, malondialdehyde (MDA), reduced glutathione (GSH) levels, as well as superoxide dismutase (SOD) and catalase (CAT) activities.

#### Total protein

The tissue protein content was determined based on Biuret method using a commercial kit (Spectrum diagnostic, Egypt).

#### Superoxide dismutase (SOD) activity

According to the method described by Nishikimi et al. (1972), it relies on the fact that nitro-blue tetrazolium (NBT) yields a blue formazan product when reduced with NADH in the presence of phenazine methosulphate (PMS). Interestingly, the presence of SOD, a common oxygen-free radical scavenger metalloenzyme that catalyzes the disproportionation of superoxide to molecular oxygen and peroxide, inhibits this reduction. There is an inverse correlation between SOD activity and the degree of NBT reduction, as measured spectrophotometrically at 560 nm.

#### Catalase (CAT) activity

This assay was conducted according to the method described by Aebi (1984**).** The decomposition of H₂O₂ that is catalyzed by CAT could be followed spectrophotometrically at 240 nm; the concentration is decreasing over time. Absorbance readings at 10-second intervals tracked the rate of H₂O₂ decomposition, proportional to catalase activity.

#### Reduced glutathione (GSH)

This assay was carried out according to Beutler et al. (1963); it depends on the fact that GSH has SH groups, which readily reduce 5,5 dithiobis 2-nitrobenzoic acid) (DTNB), giving a yellow color. Proteins lacking sulfhydryl groups were precipitated, leaving only GSH to react with DTNB. The absorbance of this product at 412 nm was directly proportional to the GSH concentration, determined by a standard curve comparison.

#### Malondialdehyde (MDA) level

Lipid peroxidation is indicated by the thiobarbituric acid reactive substances (TBARS) assay, following a modified method established by Varshney and Kale (1990). Proteins were precipitated using trichloroacetic acid (TCA), and malondialdehyde (MDA) encountered thiobarbituric acid (TBA) in an acidic environment, resulting in the formation of a pink MDA-TBA complex that was measured optically at 532 nm. The color intensity, measured spectrophotometrically, was directly related to the concentration of MDA.

#### Nitric oxide (NO) level

Liver NO content was determined colorimetrically according to the method of Montgomery and Dymock (1961). Nitric is treated with a diazotizing reagent in acidic media to form a transient diazonium salt. This is then reacted with a coupling reagent, N-naphthyl-ethylenediamine (NEDA), to form a stable compound. The absorbance is then measured at 540 nm, which is linearly proportional to the nitric concentration in the sample.

### Quantitative polymerase chain reaction (q-PCR)

#### Total RNA isolation and reverse transcription (RT)

RNA was extracted from intestine and muscle tissues by the TRIZOL RNA extraction kit (Thermo Fisher Scientific Inc., Massachusetts, USA) following the manufacturer’s instructions. The extracted RNA in DEPC-treated water was stored in aliquots at − 80 °C. The concentration and purity of the extracted RNA were then assessed using NanoDrop 2000 (Thermo 52 Materials and Methods Scientific, USA) at the Faculty of Science at Ain Shams University.

#### Reverse transcription polymerase chain reaction (RT-PCR)

The RNA template was reverse-transcribed to create double-stranded cDNA. Reverse transcription (RT) was performed using the GScript First-Strand Synthesis Kit (GeneDireX, Taiwan) according to the manufacturer’s instructions. The RNA was reverse transcribed to cDNA using Oligo dT Primer. The reaction was incubated at 25 °C for 15 min, then 37 °C for 120 min, and followed by final extension stage at 80 °C for 5 s. The cDNA product was kept at 20 °C and was ready to be used in PCR. RT was carried out with a Biometra thermocycler.

#### Real-time PCR assay

The level of gene expression in intestine and muscle tissue was quantified by real-time PCR with specific primers for the following genes: interleukin (IL-10), transforming growth factor-β (TGF-β), tumor necrosis factor (TNFα), fibronectin (FN1), and glyceraldehyde 3-phosphate dehydrogenase (GAPDH) as a housekeeping gene (Table 1). The primers were obtained from Macrogen Inc. (Seoul, South Korea), and all PCR reactions were performed using Cellixiza SYBR Green qPCR Master Mix (CellixBio, Hyderabad, India) in the Mx3005P QPCR System (Agilent Technologies Company, Santa Clara, California) according to the manufacturer’s instructions.

PCR amplifications were performed as follows: initial denaturation at 95 °C for 15 min, 40 cycles of denaturation at 95 °C for 30 s, annealing at 60 °C for 30 s, and extension at 72 °C for 45 s. The fold change in the target genes was normalized to GAPDH and calculated using the comparative Ct (2 − ΔΔCt) method (Schmittgen and Livak 2008).

**Table 1:** Specific primers used in q-RT PCR for the investigated genes

Gene	Primer Sequence (5’−3’)	GC%	Tm ºC
Fibronectin (FN1)	F: CCCCAGGTGTGATCTACGAG R: CGCTGGTGGTGAAGTCAAAG	55% 45%	68 64
Transforming Growth Factor Beta (TGF-Β1)	F: CTAATGGTGGACCGCAACAAC R: TAACGCCAGGAATTGTTGCTAT	47.6% 45.5%	62 64
Tumor Necrosis Factor-α (TNFα)	F: CAGGCGGTGCCTATGTCT R: CGATCACCCCGAAGTTCAGTAG	61.1% 50%	68 66
Interleukin 10 (IL-10)	F: GGGAGAACCTGAAGACCCTCA R: TGCTCTTGTTTTCACAGGGAAG	52.4% 40.9%	64 62
GAPDH	F: ACCACAGTCCATGCCATCAC R: TCCACCACCCTGTTGCTGTA	55% 60%	62 64

### Statistical analysis

Using Graph Pad Prism (version 8.0.2) for Windows software, intra- and intergroup statistical analyses were performed with one-way ANOVA, and then the Tukey-Kramer post-test for multiple comparisons was used to compare the different groups.

## Results

### Phytochemical constituents of S. officinalis leave extract

**Fig. 2:**
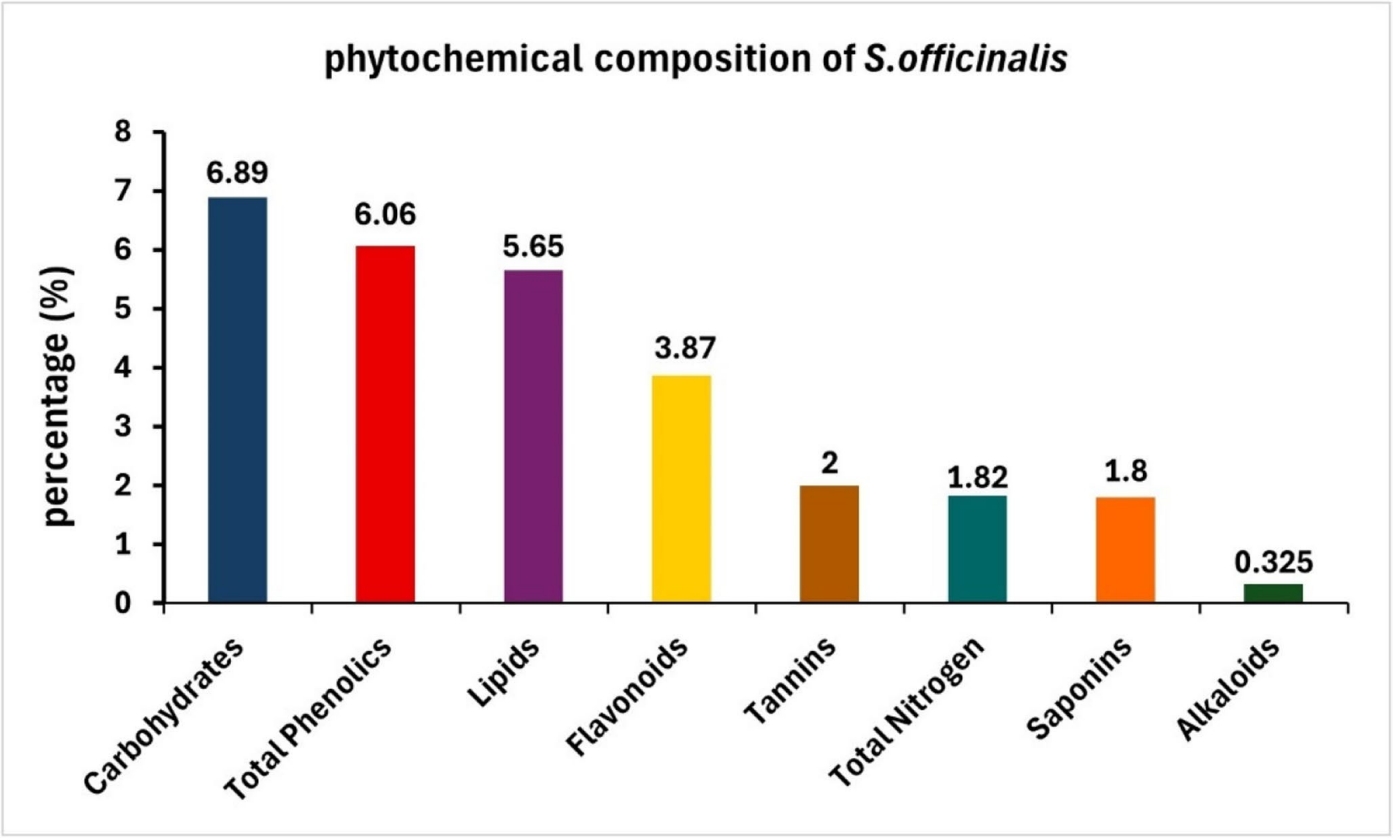
Phytochemical composition of Salvia officinalis extract. The bar graph illustrates the percentage content of various bioactive constituents

The results of the phytochemical composition) of *S. officinalis* indicated that the total phenolic compounds, flavonoids, alkaloids, tannins, saponins, carbohydrates, lipids, and total nitrogen contents in the *S. officinalis* extract were 6.06%, 3.87%, 0.325%, 2%, 1.8%, 6.89%, 5.65%, and 1.82%, respectively (Fig. 2).

### T. spiralis adult and larvae burden

A significant reduction in the mean number of *T. spiralis* adults was noted in the treated groups compared with the positive control group. Mice treated with albendazole showed the highest significant reduction, followed by combined therapy with albendazole and *S. officinalis* extract and then *S. officinalis* extract-treated groups (97.3%, 91.9%, and 80%, respectively). About the number of larvae, the mean number of total larvae in the muscles of the treated groups was significantly lower than that of the positive control group. Albendazole and *S. officinalis* treatments showed reduction percentages of 74.19% and 60%, respectively, while the group treated with both albendazole and *S. officinalis* extract showed the most reduction percentage (81.4%) (Table 2).

**Table 2:** Efficacy (as percentage reduction) of albendazole, *S. officinalis*, albendazole and *S. officinalis* against adult and encysted larvae of *T. spiralis*

	Intestinal worm	Count/mouse		Muscle larval	Count/mouse
Studied groups	Mean ± SD (range)		Efficacy (% reduction)	Mean ± SD (range)	Efficacy (% reduction)
Group (3) (Positive control)	74.50 ± 48.23 (36–105)			1283.33 ± 670.54 (914–2622)	
Group (4)(Albendazole)	2 ± 1** (1–3)		97.3% (*P* < 0.01)	331.25 ± 60.67*(258–398)	74.19% (*P* < 0.05)
Group (5) (*S. officinalis*)	14.80 ± 5.89* (9–22)		80%(*P* < 0.05)	511.75 ± 22.69* (480–530)	60% (*P* < 0.05)
Group (6) (Albendazole & *S. officinalis*)	6.00 ± 2.94* (2–9)		91.9% (*P* < 0.05)	237.75 ± 76.29** (137–310)	81.4% (*P* < 0.01)

Values expressed as mean percentage ± SD, *P* < 0.05 = * significant, **P < 0.01 = highly significant (Significant difference compared with infected control group)

### Histopathological findings

#### Small intestine (Fig. 3) (Tables 3 and 4)

**Fig. 3:**
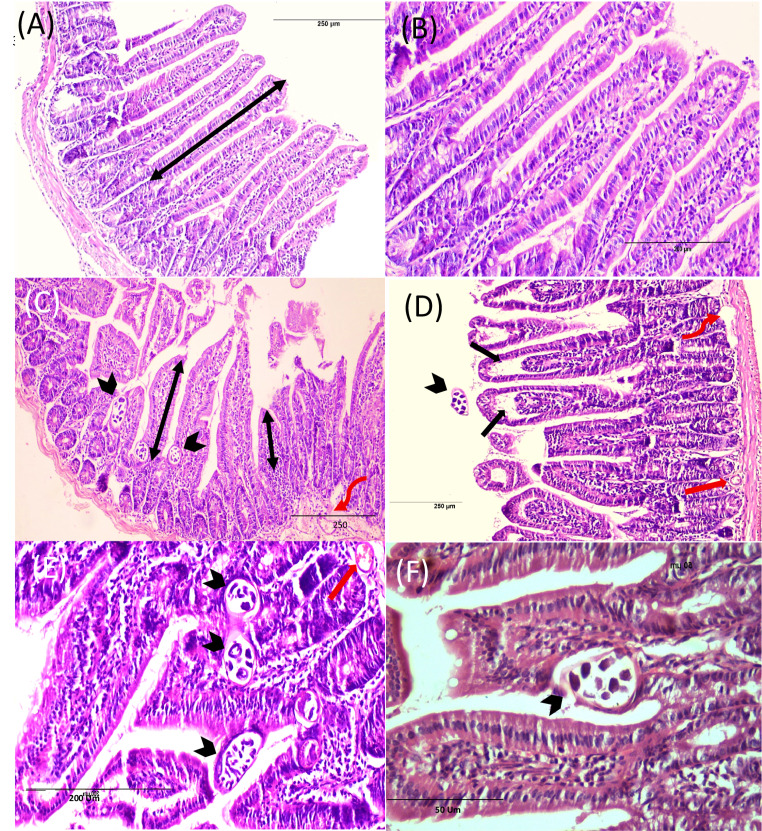

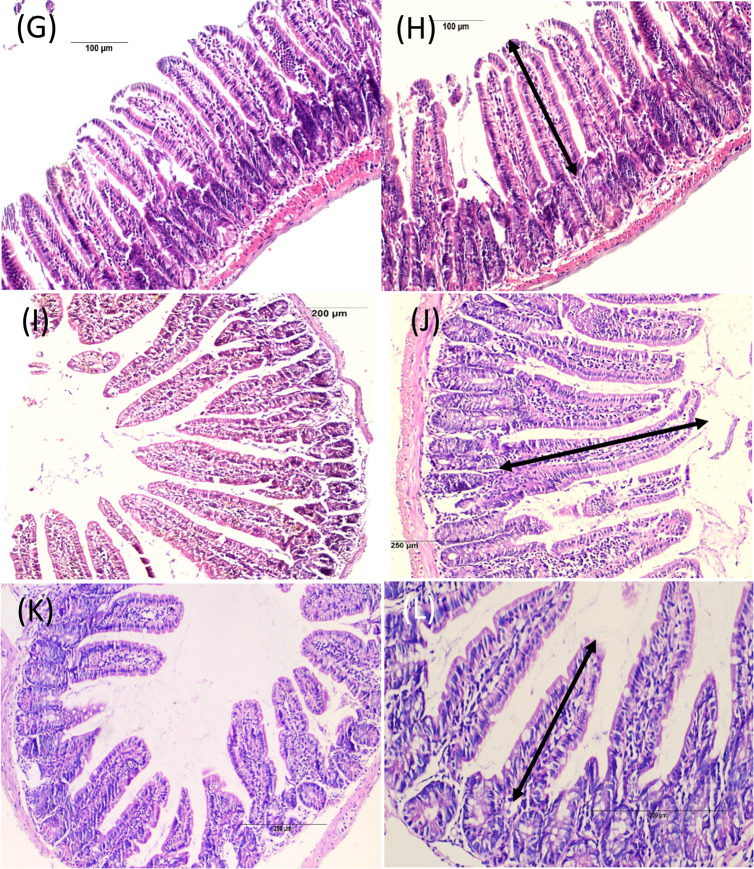
Intestinal phase of *T. spiralis* infected mice of different study groups at 7-day p.i. stained by H & E. (**A**&**B**) Non infected group with normal villous architecture. (**C**-**F**) Infected non-treated group showing flattening of villi and widening of its core by oedema (black arrows), dense chronic inflammatory cellular infiltration (score 3), in the core of the villi, submucosal edema (red wavy arrows), blood vessels congestion (red arrows) and sections of *T. spiralis* adults are seen intramucosal and in the lumen (arrow heads), Crypts hyperplasia (score 3), villus atrophy (score 2.83), increase villous/crypt ratio (2.067) and goblet cell hyperplasia (score 2) are also noted. (**G**&**H**) ALB-treated group showing more villous preservation and mild inflammatory infiltrate (score 1.6) with villous/crypt ratio of 3.15. (**I**&**J**)* S. officinalis* treated group with best improvement obtained, with greatly improved villus height (two headed arrows) and well-preserved villi with noticeable reduction in the intensity of the inflammatory infiltrate (score 1.5). (**K**&**L**) Combination therapy treated group showed an improvement in villous pattern and moderate inflammatory infiltrate (score 1.83)

The histopathological assessment of intestinal tissues from the negative control group revealed a normal architecture of the four layers: mucosa, submucosa, muscularis, and serosa (Fig. 3, A&B). In contrast, intestinal tissue sections from the positive control group (Fig. 3, C-F) showed histological indications of inflammation within the mucosal and submucosal layers and the core of the villi. It showed some flattened leaf-shaped, degenerated intestinal villi with distortion of cells on the tips with edema in some areas. Some areas exhibited a fusion of adjacent villi and complete villous atrophy. A reduction in villus height and increases in Lieberkühn crypt depth were also noted with hyperplasia of the crypts (enlargement), and the lamina propria appears densely packed with inflammatory cells. The muscle layer displayed cytoplasmic vacuolation in some areas. Also, parasite sections were observed among the mucosal layer with goblet cell hyperplasia in some places.

Regarding the treated groups (Fig. 3, G-L**)**, there was a noticeable improvement in histopathological findings noted as a reduction in the inflammation, an improvement in the intestinal architecture and villi length, along with reduced edema and absence of worm sections; the best improvement was obtained in the *S. officinalis*-treated subgroup, with greatly improved villus height.

**Table 3:** Semi quantitative scoring of histopathological changes during the intestinal phase in the studied groups

Studied groups	Inflammatory infiltration	Goblet cells	Crypt Hyperplasia	Villus Atrophy
Group (1) (-ve control)	0.5	0.5	0	0.5
Group (2) -ve control *(S. officinalis*)	0.5	0.5	0	0.5
Group (3) (positive control)	3	2	3	2.83
Group (4) (Albendazole)	1.6^a^	1.66	2.16^a^	1^a^
Group (5) (*S. officinalis*)	1.5^a^	1.16^c^	2^a^	1^a^
Group (6) (Albendazole & *S. officinalis*)	1.83^b^	1.5	2^a^	1^a^

(0) means no change, (1) mild, (2) moderate, and (3) severe changes. Different superscript letters indicate statistically significant differences between groups, a: *P* < 0.0001, b: *P* < 0.001, c: *P* < 0.01, “Group (2) showed no histopathological changes compared to the negative control”

**Table 4:** Morphometric measurements of intestinal villi and crypts in different experimental groups

Studied groups	Villus height Mean ± SD	Crypts depth Mean ± SD	Villus height to crypts depth ratio
Group (1) (-ve control)	363.73 + 33.61	78.91 + 12.62	4.6
Group (2) -vecontrol *(S. officinalis*)	369.14 + 25.62	79.74 + 15.54	4.62
Group (3) (positive control)	259.01 + 53.27	125.288 + 17.33	2.067
Group (4) (Albendazole)	291.85 + 57.14	92.429 + 13.55	3.15
Group (5) (*S. officinalis*)	361.52 + 88.16	88.348 + 14.23	4.09
Group (6) (Albendazole & *S. officinalis*)	342.1 + 72.6	106.36 + 22.1	3.2

The table shows villus height, crypt depth, and the calculated villus height to crypt depth ratio in micrometers (µm). Values are expressed as mean ± standard deviation (SD), “Group (2) showed no histopathological changes compared to the negative control”

#### Diaphragm (Fig. 4)

**Fig. 4:**
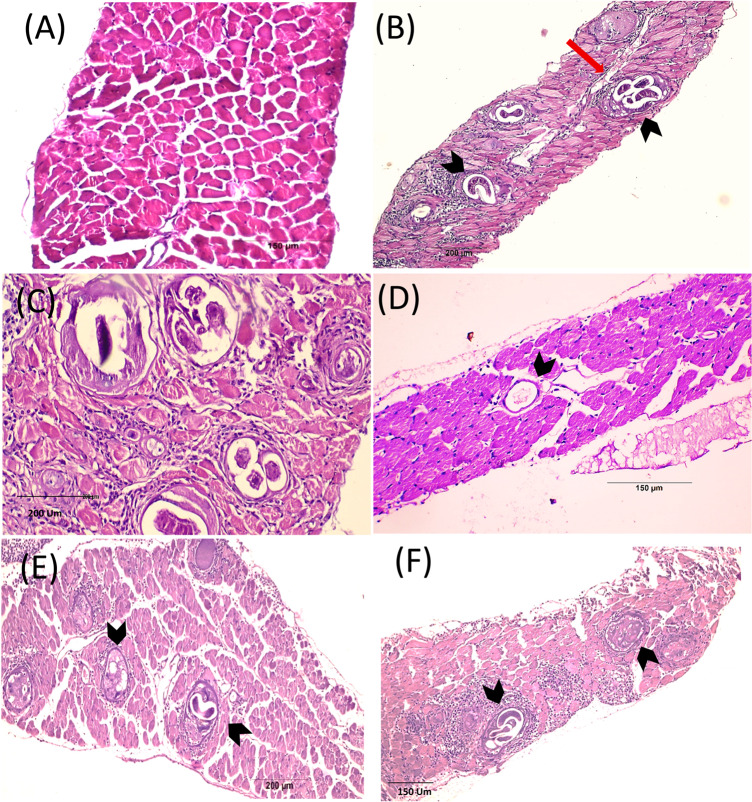

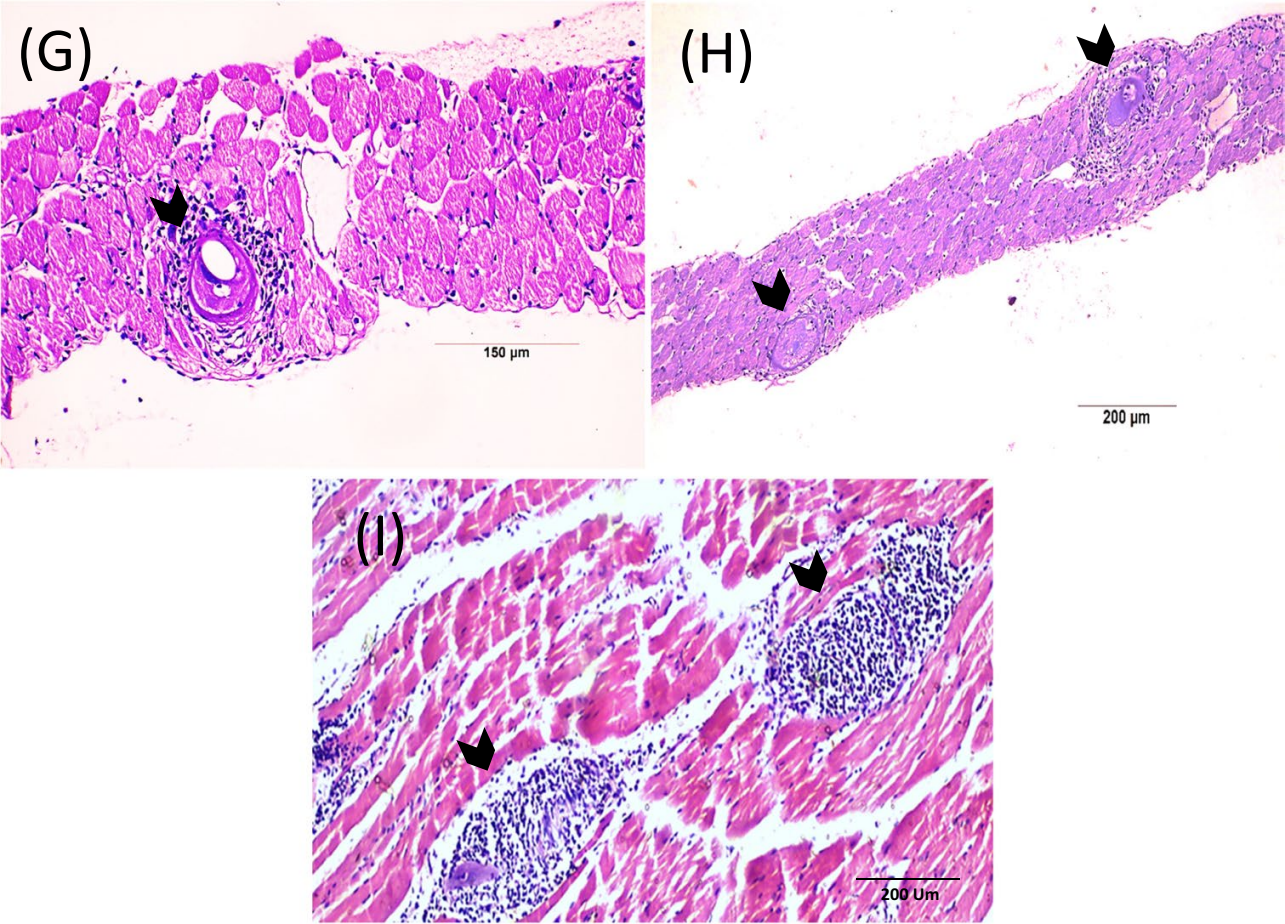
Muscle phase of *T. spiralis* infected mice of different study groups at 37-day p.i. showing diaphragm sections stained by H&E. (**A**) Control non-infected group with normal architecture of diaphragm. (**B**&**C**) Infected non-treated group showing intense inflammatory infiltrate all over the diaphragm with multiple encysted *T. spiralis* larvae (arrow heads), embedded in the diaphragm with thick capsule around the larvae and granuloma formation with degeneration of muscle fibers and increased fibrous tissue depositions (red arrow). (**D**&**E**) ALB-treated group showing less depositions of the encysted capsule of *T. spiralis* and moderate inflammatory infiltrate associated with marked lysis of the larva. (**F**&**G**) *S. officinalis* treated group showing moderate number of *T. spiralis* larva with degenerated capsule surrounded by moderate inflammatory cellular infiltrates. (**H**&**I**) Combination therapy treated group showing deteriorated cysts replaced by a moderate inflammatory infiltrates and smaller cysts with degenerated larva

Histopathological analysis of diaphragm sections from the normal control group revealed typical diaphragm structure (Fig. 4, A). In contrast, the infected control group exhibited numerous encysted *T. spiralis* larvae with intact capsules, dispersed among degenerating muscle fibers, accompanied by chronic inflammatory cells (Fig. 4, B&C). The treated groups (Fig. 4, D-I) showed marked histopathological improvement compared to the infected control group, with fewer cysts exhibiting degenerated capsules and localized pericapsular inflammation. The *S. officinalis* and albendazole combination treatment yielded the most significant improvement, displaying the fewest cysts and degenerated larval capsules with disrupted internal structures and inflammatory cell infiltration. In some areas, encysted larvae were absent, replaced by eosinophilic exudates.

### Oxidative stress markers

#### Day 7 post-infection

**Fig. 5:**
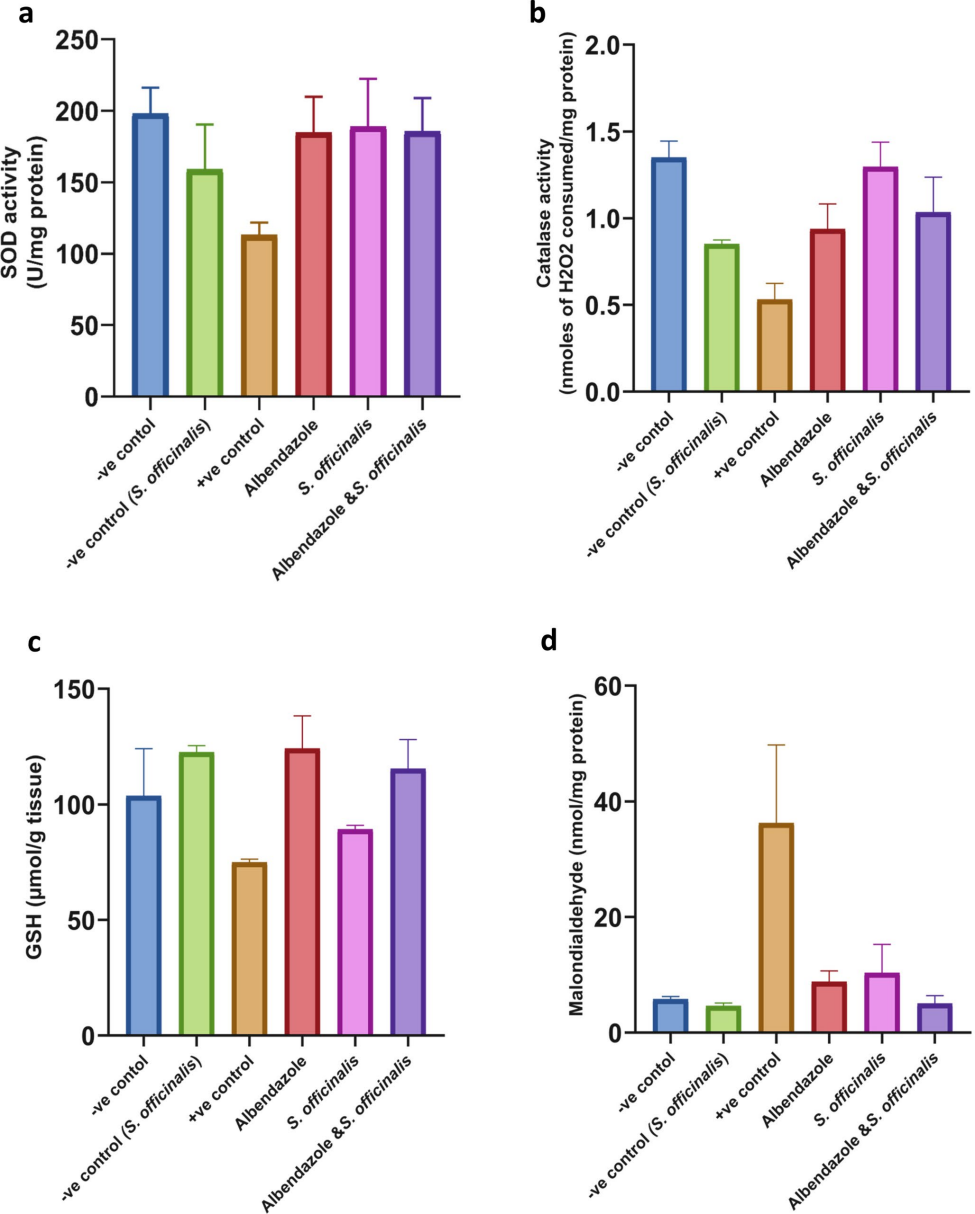

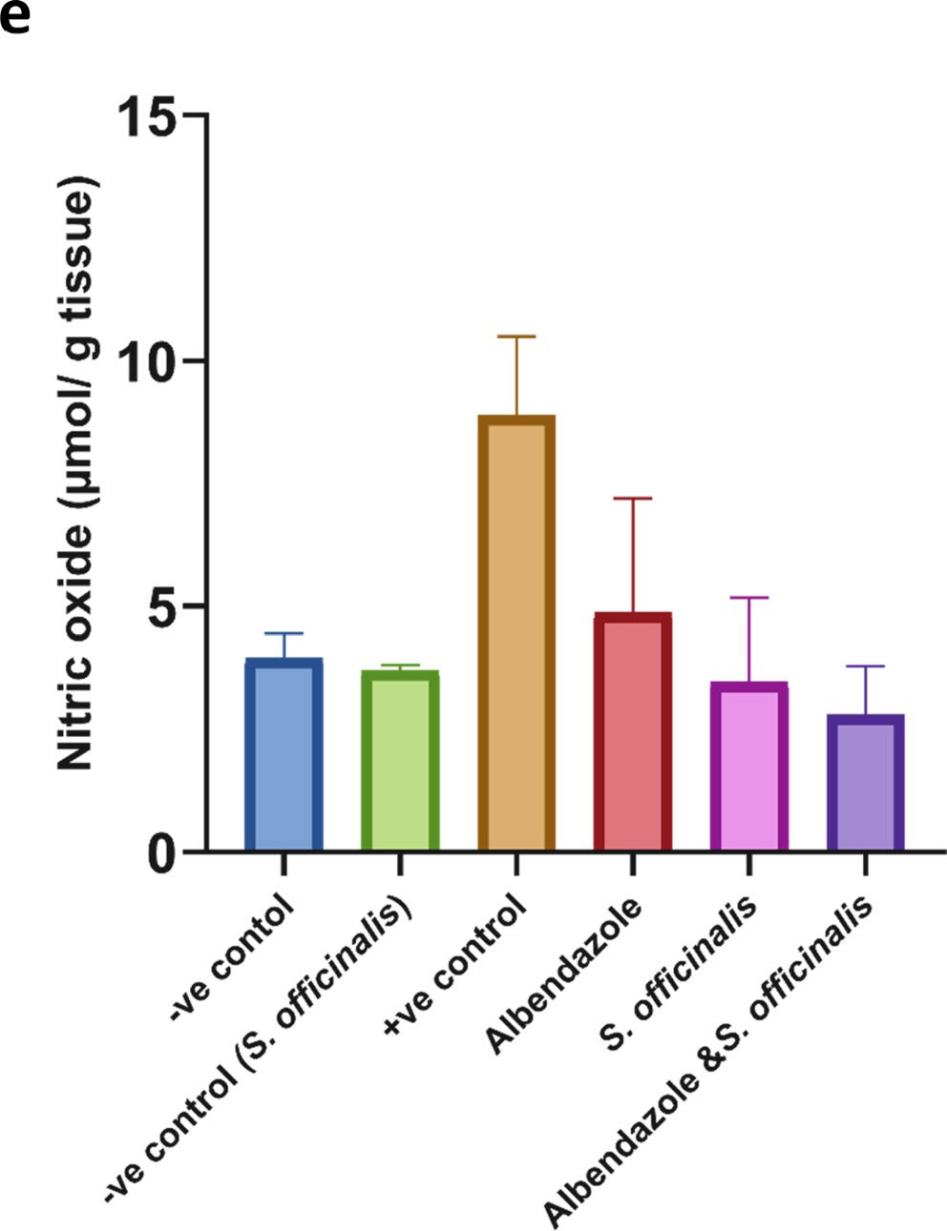
The effects of the different treatments on various oxidative stress markers in Liver tissues at day 7 p.i. (**a**) superoxide dismutase (**b**) catalase (CAT), (**c**) reduced glutathione (GSH), (**d**) malondialdehyde (MDA), and (**e**) nitric oxide (NO). The data were expressed as mean ± SD

Evaluation of oxidative stress markers (mean ± SD) (in liver tissues) among healthy control, infected control, and treated groups was noticed. The level of SOD activity was significantly decreased in the positive control group compared to the negative control group (*P* < 0.01). In the treated groups, SOD activity was significantly higher (*p* < 0.01) in mice given albendazole, *S. officinalis* extract, or combined treatment with albendazole and *S. officinalis* extract compared to *T. spiralis*-infected mice (Fig. 5.a), reaching levels close to those of the normal control group (*P* = 0.98–0.99).

Treating the infected mice with *S. officinalis* extract alone displayed a highly significant (*P* < 0.0001) increase in the catalase activity compared to the infected group, which highly decreased in comparison to the healthy group (*P* < 0.0001), restoring CAT activity nearly to the levels observed in the normal control group, with no statistically significant difference (*P* = 0.9). Also, treating mice with albendazole combined treatment increased significantly (*P* < 0.001, *P* < 0.05) the catalase activity compared to the infected group (Fig. 5.b). While combined treatment showed a non-significant change when compared to the negative control (*P* = 0.11), albendazole has a significant difference compared to the normal group (*P* > 0.01).

The infected group showed a moderate decrease in GSH level relative to the negative control, while the albendazole-treated group showed the highest level of GSH with the highest significant increase (*P* < 0.001) compared to the infected group, exceeding that of the negative control. Upon treating the infected mice with combined treatment, the GSH content increased significantly (*P* < 0.01) compared to the infected group, while treating mice with *S. officinalis* extract alone increased GSH level insignificantly (Fig. 5.c).

Regarding MDA tissue levels, there was a significantly higher value in the infected control group compared to the non-infected control group (*P* < 0.0001), signifying the oxidative stress during *T. spiralis* infection. However, its level decreased in all treated groups with a highly significant difference compared to the infected control group (*P* < 0.0001), which has no statistically meaningful deviation (*P* = 0.7–0.9) from the normal control (Fig. 5.d).

The data demonstrate that *T. spiralis* infection significantly raised the level of NO in tissue (*P* < 0.01), while the *S. officinalis* extract and the combined treatment have significantly decreased it (*P* < 0.001); also, the albendazole group showed the lowest significant decrease in NO level (*P* < 0.01) relative to the infected group (Fig. 5.e), bringing NO level close to normal baseline levels with no significant variations.

#### Day 37 post infection

**Fig. 6:**
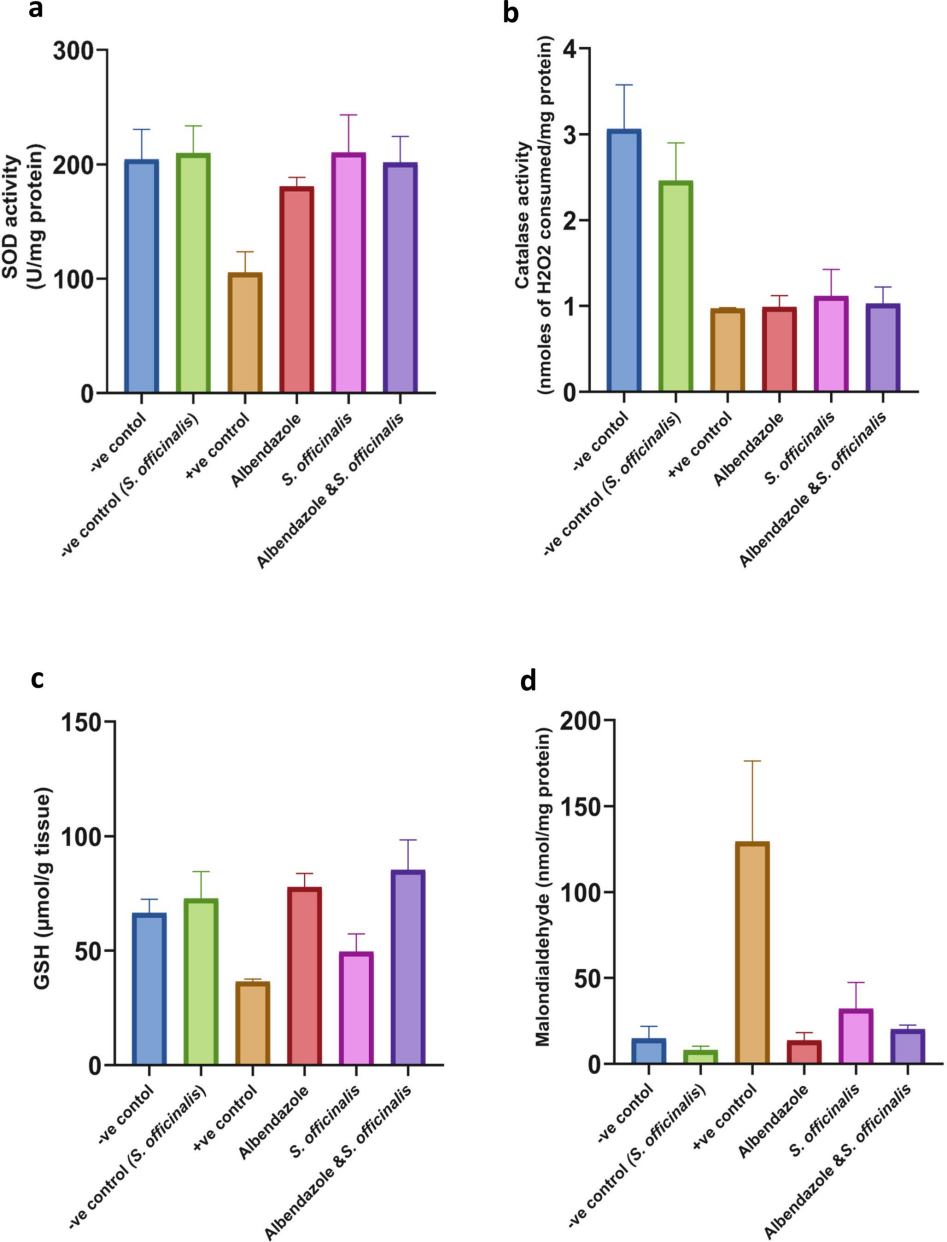

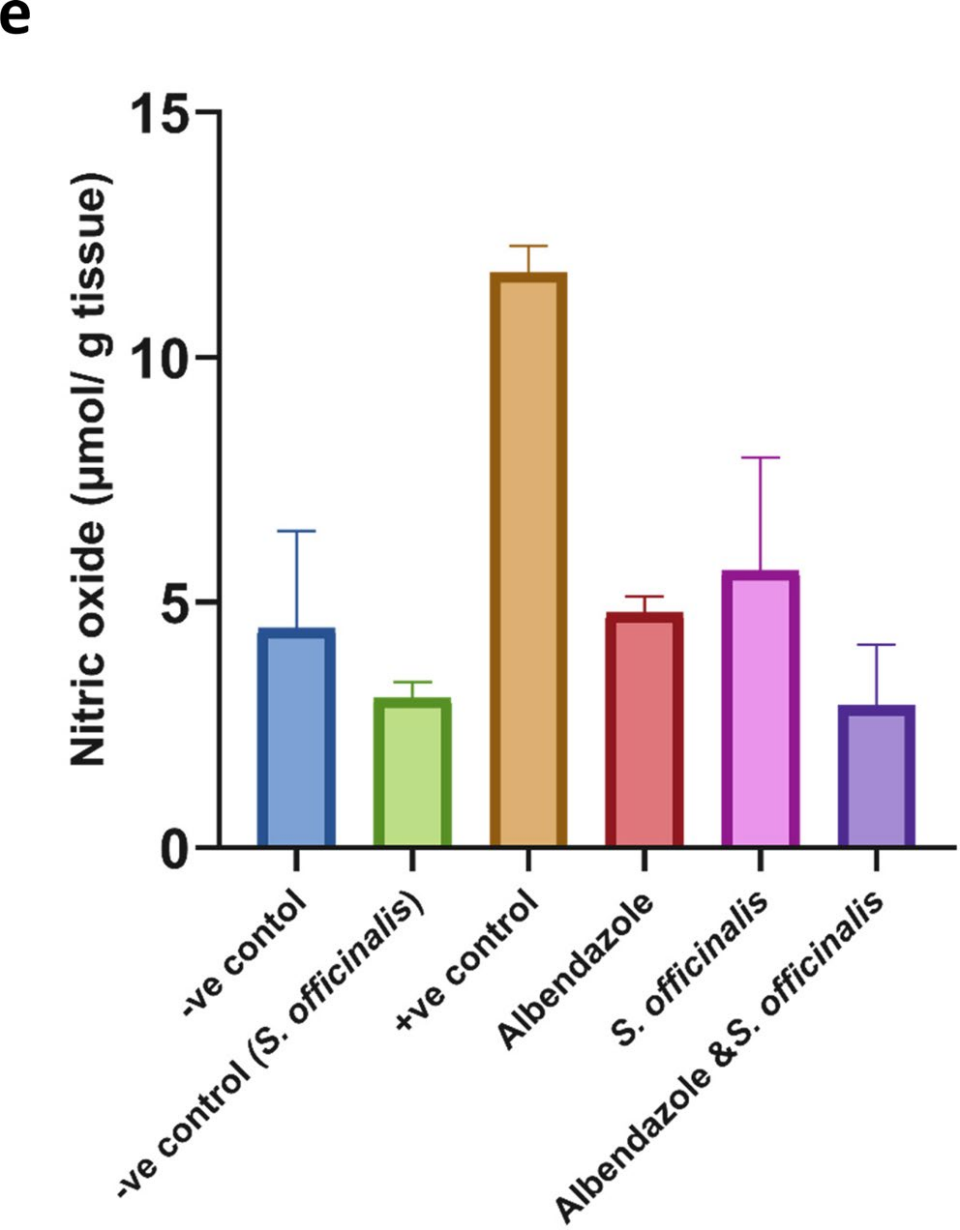
The effects of the different treatments on various oxidative stress markers in Liver tissues at day 37 p.i. (**a**) superoxide dismutase (**b**) catalase (CAT), (**c**) reduced glutathione (GSH), (**d**) malondialdehyde (MDA), and (**e**) nitric oxide (NO). The data were expressed as mean ± SD.3

Concerning the SOD activity in liver tissues, there are highly significant increases (*p* < 0.001) in the mice given *S. officinalis* extract and combined treatment compared to the albendazole group compared to the positive infected mice, which showed a significant decrease in SOD level (*p* < 0.001), restoring SOD activity nearly to the levels observed in the normal control group (*P* = 0.8–0.9) (Fig. 6.a).

In the infected control group, the catalase activity decreased significantly compared to the negative control group (*p* < 0.0001), while treating the infected mice with albendazole, *S. officinalis* extract, and combined treatment displayed a non-significant change in catalase level compared to the infected group (Fig. 6.b).

Also, treated groups with albendazole combined treatment showed a significant increase (*P* < 0.005, *P* < 0.001) in GSH level compared to the infected group, which decreased significantly (*P* < 0.01) in reference to healthy mice. while *S. officinalis* extract treatment alone did not reach statistical significance (Fig. 6.c).

A marked elevation in MDA level was observed after *T. spiralis* infection (*P* < 0.0001), while albendazole, *S. officinalis* extract, and combined treatment have decreased the MDA levels in mice groups with a highly significant difference compared to the infected one (*P* < 0.0001), normalizing MDA to levels closely resembling those of the normal control group, showing no significant difference from the control (*P* = 0.7–0.9) (Fig. 6.d) infection with *T. spiralis* significantly (*P* < 0.0001) elevated NO level. While the administration of albendazole, *S. officinalis* extract, and combined therapy caused a highly significant decrease in NO level (*P* < 0.0001, *P* < 0.0005, and *P* < 0.001, respectively) compared to the infected group, reestablishing NO to near-normal control levels with no significant difference detected (*P* = 0.7–0.9) (Fig. 6.e).

### Real-time PCR analysis of differentially expressed genes

#### In intestinal tissues (Table 5; Fig. 7.A)

The fold change analysis of gene expression in intestinal tissue revealed significant alterations among experimental groups. FN1 expression was markedly elevated in the infected control group, almost by 24-fold, indicating pronounced fibrosis and tissue remodeling. Treatment with albendazole significantly reduced FN1 expression by 9-fold compared to the infected group (*P* < 0.001), demonstrating its efficacy in mitigating muscle damage, whereas *S. officinalis* extract led to a marked downregulation by 44-fold (*P* < 0.001), reflecting moderate antifibrotic effects. The combination therapy also showed a reduction (almost by 9-fold) but remained slightly elevated compared to untreated controls, indicating an enhanced therapeutic effect.

TNFα, a key pro-inflammatory cytokine, exhibited a significant upregulation in the infected control group by 32-fold compared to the negative control group, reinforcing the inflammatory response induced by *T. spiralis*. Albendazole alone had a minimal effect, which decreased expression by 76-fold, while *S. officinalis* extract significantly downregulated TNFα expression (*P* < 0.0001). Combination therapy also resulted in a significant reduction (*P* < 0.0001).

TGF-β, a crucial fibrosis-associated marker, was upregulated in the infected control group by 175-fold. Albendazole treatment slightly decreased TGF-β expression by 26-fold compared to the infected group, while *S. officinalis* extract monotherapy nonsignificantly reduced TGF-β by almost 70-fold. The combination treatment also showed a partial reduction by almost 30-fold, suggesting a regulatory role of *S. officinalis* in fibrosis control.

Compared to the uninfected group, IL-10, an anti-inflammatory cytokine, was significantly upregulated in the infected control group (78-fold). Albendazole, *S. officinalis* extract, and their combination moderately reduced IL-10 levels by 39-fold, 45.8-fold, and 216.6-fold, respectively, compared to the infected group (*P* < 0.01).

**Table 5:** The mean and the standard error of mean fold change values of the expression of some genes in the intestinal tissue

Group Gene	Group (1) -ve Control	Group (3) + ve control	Group (4) (Albendazole)	Group (5) (S.officinalis)	Group (6) (Albendazole & S.officinalis)
FN1	1.00 ± 0.10	23.87 ± 4.9	2.66 ± 1.3	0.54 ± 0.09	2.63 ± 1.7
TNF-α	1.00 ± 0.12	32.34 ± 4.075	0.42 ± 0.36	0.039 ± 0.02	0.066 ± 0.03
TGF-β	1.00 ± 0.10	175.1 ± 140.1	6.6 ± 3.3	2.5 ± 0.4	5.8 ± 3.7
IL-10	1.00 ± 0.11	78.19 ± 37.9	2.01 ± 1.6	1.73 ± 1.37	0.36 ± 0.09

The results are expressed as mean ± S.E.M. The data were analyzed with one way ANOVA, and then for multiple comparisons, Tukey test was applied

**Fig. 7:**
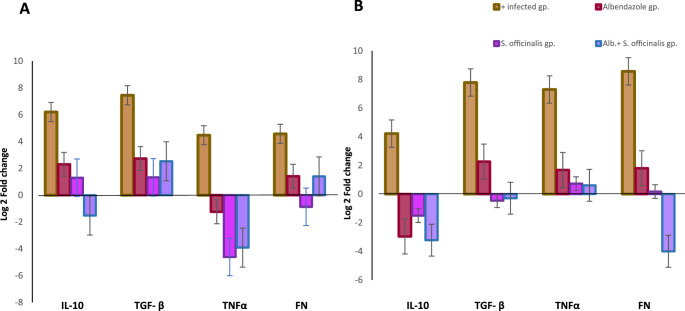
Graph showing the log2 fold change of FN1, TNF-α, TGF-β, and IL-10 (**A**) in intestinal tissues 7 days p.i (**B**) in muscle tissues 37 days p.i across experimental groups

#### In muscle tissues (Table 6; Fig. 7.B)

In muscle tissue, infection significantly upregulated FN1 expression by 379-fold, suggesting substantial fibrosis and extracellular matrix remodeling. Albendazole moderately reduced this expression by 111-fold (*P* < 0.001), indicating efficacy against infection-induced muscle damage. *S. officinalis* extract was more effective, reducing FN1 expression by 343.6-fold (*P* < 0.001). Combination therapy resulted in the greatest reduction, 393.7-fold (*P* < 0.001), highlighting potential synergistic benefits.

Similarly, TNFα expression was markedly elevated in the infected control group (157.5-fold). Albendazole had a limited effect, decreasing TNFα expression by approximately 51-fold, while *S. officinalis* extract suppressed it more effectively by 95-fold (*P* < 0.0001). The combination therapy further reduced TNFα expression by 105-fold (*P* < 0.0001), demonstrating enhanced anti-inflammatory activity.

In the infected control group, TGF-β, a key fibrosis-related cytokine, increased dramatically by approximately 192-fold. Albendazole, *S. officinalis* extract, and their combination partially reduced TGF-β levels by 31.4-fold (*P* < 0.005), 266-fold, and 240-fold (*P* < 0.001), respectively.

The infected control group also showed an 18.6-fold elevation in IL-10, an important anti-inflammatory cytokine. Albendazole non-significantly reduced IL-10 by 155-fold. *S. officinalis* extract maintained a moderate anti-inflammatory response, decreasing IL-10 by 53-fold. The combination therapy further suppressed IL-10 by 186-fold, suggesting a potential immunoregulatory effect.

**Table 6:** The mean and the standard error of mean fold change values of the expression of some genes in the muscle tissue

Group Gene	Group (1) -ve Control	Group (3) Positive control	Group (4) (Albendazole)	Group (5) (S.officinalis)	Group (6) (Albendazole &S.officinalis)
FN1	1.00 ± 0.10	378.8 ± 133.2	3.4 ± 2.9	1.12 ± 0.5	0.96 ± 0.45
TNFα	1.00 ± 0.09	157.5 ± 11.5	3.1 ± 1.1	1.65 ± 0.7	1.5 ± 0.37
TGF-β	1.00 ± 0.09	192.2 ± 72.8	6.1 ± 5.7	0.72 ± 0.31	0.82 ± 0.5
IL-10	1.00 ± 0.11	18.63 ± 13.8	0.12 ± 0.05	0.35 ± 0.2	0.1 ± 0.08

The results are expressed as mean ± S.E.M. The data were analyzed with one way ANOVA, and then for multiple comparisons, Tukey test was applied

## Discussion

Trichinellosis, a global public health concern, is difficult to treat due to the immune response, diverse tissue involvement, and effector cells during different infection phases (Hamed et al. 2022). While albendazole is effective in the intestinal phase, its bioavailability limits its efficacy against the muscular phase (Attia et al. 2015). Given that 80% of people in underdeveloped countries rely on traditional medicine (El-Wakil et al. 2022; El-Wakil et al. 2023) and natural foods are rich in bioactive molecules (Bruno et al. 2021), there is a need for an alternative, safe, and effective herbal medicine to treat both intestinal and muscular stages of *T. spiralis* (Shalaby et al. 2010).

Albendazole is associated with several adverse reactions, including encephalitis, convulsions, and severe drug eruptions (Yadav and Temjenmongla 2012; El-Wakil et al. 2023). This study, therefore, evaluated the efficacy of *Salvia officinalis* (sage) leaf extract against different *T. spiralis* stages in mice, comparing it to albendazole. Phytochemical screening is useful for examining the secondary metabolites of bioactive plants (Farag et al. 2020). The efficacy of *S. officinalis* against *T. spiralis* is likely due to its phytochemical composition. Sage contains bioactive constituents, such as rosmarinic acid, carnosic acid, and essential oils, with documented antimicrobial and anti-inflammatory activities (Hamidpour et al. 2014).

Sage leaf contains various compounds, including acids, glycosides, nicotinamide, flavones, flavonoids, niacin, and estrogenic compounds (Saleem et al. 2010). Our analysis revealed significant amounts of terpenoids, steroids, phenols, flavonoids, tannins, saponins, carbohydrates, and lipids, all with distinct pharmacological properties. Tannins may induce antiparasitic effects by interacting with parasite surface proteins, leading to physiological dysfunction and mortality (El-Samee et al. 2024). These findings support the antiparasitic potential of medicinal plants, especially those abundant in bioactive compounds like flavonoids, terpenoids, and phenolic acids (Ranasinghe et al. 2023). Also, in vitro, and in vivo studies on the nematocidal effects of the essential oil (monoterpinoides) were extensively carried out on root-knot nematode *Meloidogyne incognita* (Echeverrigaray et al. 2010).

The antiparasitic effect of *S. officinalis* has been previously shown on *Syphacia obvelata*,* Aspiculoris tetrapetra* and *Hymenolepis nana* parasites (Amirmohammadi et al. 2014), *Leishmania major* (Nikmehr et al. 2014), *Cryptosporidium parvum* (Abouelsoued et al. 2020; Al-Dulaimi et al. 2021), *Eimeria tenella* (Sidiropoulou et al. 2022) and *Ichthyophthirius multifiliis* (Özil 2023).

In this study, our finding revealed a significant reduction in the adult worm and larvae counts of *T. spiralis* in albendazole (97.3%, 74.19%) and *S. officinalis* extract (80%, 60%*).* Adding the dose of *S. officinalis* (250 mg/kg) to the half dose of albendazole (25 mg/kg) treatment led to the highest reduction rate (91.9%, 81.4%) compared with the infected untreated group, showing a greater effect than albendazole alone in the muscle phase of *T. spiralis*.

Although albendazole monotherapy exhibited a slightly higher reduction rate of adult worms in the intestinal phase (97.3%) compared to the combination therapy (91.9%), the latter was more effective in the muscular phase. This difference in intestinal efficacy may be attributed to *S. officinalis*’s potential induction of intestinal CYP2C19 and inhibition of CYP3A4, impacting albendazole’s first-pass metabolism (Hellum et al. 2007). Since albendazole requires CYP3A-mediated activation to albendazole sulfoxide in enterocytes (Molina et al. 2007), S. *officinalis* modulation could reduce local sulfoxide formation, slightly decreasing efficacy against adult worms in the gut. Furthermore, compounds in *S. officinalis*, such as rosmarinic acid and thujone, possess anti-inflammatory and immunomodulatory effects. By potentially diminishing the essential inflammatory response in the gut needed for expelling adult *T. spiralis*, the plant extract may inadvertently reduce the host’s natural worm-clearing mechanisms, thus slightly lowering the drug’s intestinal efficacy.

In the muscular phase, the plant extract may lower the presystemic conversion of albendazole in the intestine, leading to increased delivery to the liver. The extract’s partial CYP3A4 inhibition in the liver prolongs the drug’s half-life, enhancing its effect against muscular larvae. Additionally, the extract’s anti-inflammatory, antioxidant, and immunomodulatory properties may reduce muscle damage and improve parasite clearance during this phase.

Several studies report varying effectiveness of different treatments against *T. spiralis*. Albendazole reduced worm counts by 90.91% in one study (Fadl et al. 2020), while the combination of albendazole and arginine resulted in a 72.7% reduction. Salama et al. (2022) found that *Zingiber officinale* and *Cinnamomum zeylanicum* reduced adult worm counts by 64.5% and 68% and larvae counts by 50.8% and 54.6%, respectively, compared to albendazole’s 93.5% and 90.6% reduction rates. Curcumin (300 mg/kg) reduced intestinal worm counts by 65.35%, while albendazole reduced them by 90.78% (Hamed et al. 2022), curcumin was more effective than albendazole alone in reducing muscular larvae (70.67% vs. 56.49%). Ashoush et al. (2024) demonstrated that ellagic acid reduced adult worm counts by 62%, while albendazole reduced them by 94%.

Histopathological examination revealed marked inflammation in intestinal and diaphragm tissues due to *T. spiralis*, consistent with prior research (Saracino et al. 2020; Elmehy et al. 2021; Abd-ELrahman et al. 2024; Saleh et al. 2024; Hamed et al. 2022; Salama et al. 2022). This study observed that treatments enhanced histopathological changes in both trichinosis phases, significantly reducing inflammatory cells and restoring intestinal villi architecture compared to the infected control group. In the muscular phase, treatments reduced larvae and cellular infiltrates while increasing regenerative muscles. These findings suggest that *S. officinalis* possesses anti-inflammatory properties, as reported in previous pharmacological studies (Abad et al. 2011; Mansourabadi et al. 2016; Rodrigues et al. 2012; Ghorbani and Esmaeilizadeh 2017).

During *T. spiralis* infection, both the parasite and host generate large amounts of reactive oxygen species (ROS) and free radicals, leading to significant oxidative stress that contributes to tissue damage, especially in skeletal muscles (Othman et al. 2016; Elgendy et al. 2020). ROS can induce cellular injury through covalent binding, lipid peroxidation, DNA strand breaks, and stimulation of fibrosis (Gao et al. 2011; Shahrzad et al. 2014). In response, antioxidant enzyme activities such as superoxide dismutase (SOD), catalase (CAT), and glutathione (GSH) increase as part of the host’s defense mechanism. These enzymes work collectively to neutralize superoxide radicals and mitigate oxidative damage (Adegbola et al. 2022; Gabrashanska et al. 2019; El**-**Hamed et al. 2022).

Nitric oxide (NO) also plays a dual role in host defense, mediating antiparasitic effects of proinflammatory cytokines (Burgner et al. 1999). However, excess NO and elevated MDA levels, indicative of lipid peroxidation, contribute to host tissue injury. *T. spiralis* infection is associated with decreased levels of GSH, SOD, and CAT, accompanied by elevated MDA and NO levels (Elgendy et al. 2020; Hamed et al. 2022; Salama et al. 2023; Khedr et al. 2024; Ashoush et al. 2024; Albogami 2023; Alghabban et al. 2024; Nagdy et al. 2025). These findings highlight oxidative imbalance during infection and the need for interventions that restore redox homeostasis.

Contrary to some reports that observed elevated antioxidant enzyme levels during infection (Derda et al. 2004; Gabrashanska et al. 2019), our study supports the prevailing view that *T. spiralis*-induced oxidative stress suppresses antioxidant defenses.

In this context, *S. officinalis* (sage) extract demonstrated a significant antioxidant effect, particularly in the treated infected groups and even more so in the non-infected treated group (G2). This supports the plant’s well-documented antioxidative potential, attributed to its content of flavonoids, luteolin-7-glucoside, and rosmarinic acid compounds with strong anti-inflammatory and antioxidant properties (Brindisi et al. 2020, 2021). These findings are consistent with previous studies highlighting the antioxidant capacity of *S. officinalis* (Al-Mijalli et al. 2022; Balčiūnaitienė et al. 2022; Jedidi et al. 2022; Metin et al. 2024; Amer et al. 2024).

The administration of *S. officinalis* significantly enhanced host antioxidant and immune responses, particularly through upregulation of SOD activity. This likely contributed to reduced ROS-mediated damage, improved intestinal tissue integrity, and a more effective immune response capable of limiting parasite survival. The marked reduction in MDA and NO levels, along with increased levels of SOD, CAT, and GSH, in treated groups, confirms the ability of *S. officinalis* to restore redox homeostasis and create a less favorable environment for *T. spiralis* persistence.

The findings of this study highlight the therapeutic potential of *S. officinalis* in modulating the inflammatory and fibrotic responses associated with *T. spiralis* infection. The gene expression analysis revealed significant alterations in FN1, TNFα, TGF-β, and IL-10, which play crucial roles in fibrosis, inflammation, and immune regulation. While albendazole, the standard antiparasitic drug, exhibited moderate effects in controlling inflammation, its combination with *S. officinalis* extract demonstrated superior regulatory effects, indicating a potential synergistic benefit in reducing fibrosis and inflammatory responses.

Fibronectin (FN1), an extracellular matrix glycoprotein crucial in fibrosis and tissue repair (Sofroniou and Lemmon 2025), was upregulated in infected control groups in both intestinal and muscle tissues, indicating significant tissue remodeling and fibrotic activity post-T. *spiralis* infection, particularly in muscle. This increased expression correlated with elevated MDA and NO levels, suggesting that oxidative and nitrosative damage induces ECM remodeling (Martins et al. 2021). The subsequent decline in FN1 expression following treatment supports the protective role of *S. officinalis* extract in mitigating fibrosis risk. Combination therapy further suppressed FN1 expression, indicating a stronger regulatory effect on ECM remodeling. These results align with previous findings that medicinal plants like *S. officinalis* possess collagen-modulating properties (Li et al. 2019; Wu et al. 2022), suggesting their potential as adjunct therapies for parasite-induced fibrosis (Ghorbani and Esmaeilizadeh 2017), consistent with observed fibrotic changes and ECM deposition in trichinellosis.

In the infected group, elevated TNFα indicates an acute inflammatory response to *T. spiralis* invasion, mediating tissue inflammation as reported previously (Breloer and Abraham 2017). TNFα overexpression likely contributed to ROS accumulation and elevated MDA by stimulating oxidative burst. This corresponds with decreased antioxidant enzyme activities (SOD, catalase, and GSH) and increased MDA and NO levels.

Treatment with *S. officinalis* extract significantly suppressed TNFα, with a greater effect observed in the combination therapy. This reflects the anti-inflammatory potential of *S. officinalis*, attributed to its rich content of phenolic compounds, including rosmaric acid, which may inhibit NF-kB signaling and other inflammatory pathways.

In helminth infections, TNFα serves a dual function: it facilitates the clearance of parasites by the immune system, but it can also induce tissue injury and excessive inflammation. The observed downregulation of TNFα in *S. officinalis*-treated groups may be indicative of a protective anti-inflammatory effect that promotes the integrity of the intestinal barrier and inhibits parasite invasion. However, reduced TNFα could also compromise immune clearance. Similar TNFα reductions, alongside other improvements, have been observed with albendazole and lactoferrin-loaded silver nanoparticles, as well as with eugenol, suggesting the latter’s potential as a trichinellosis treatment (Atta et al. 2024; Bakr et al. 2025).

The higher expression levels of TNFα observed during the muscle phase compared to the intestinal phase may be attributed to the chronic nature of the muscle-stage infection by *T. spiralis.* In this phase, the larvae encyst within muscle tissue, leading to prolonged antigenic stimulation and sustained inflammation. TNFα plays a key role in mediating chronic inflammation, cellular infiltration, and tissue remodeling, including fibrosis and myositis (Fabre et al. 2009). Additionally, studies have shown that the muscle stage of *Trichinella* infection is associated with a more intense and localized immune response compared to the intestinal stage (Beiting et al. 2004; Bruschi and Chiumiento 2011**)**, which may explain the higher TNF-α expression. The treatments may have further enhanced immune recognition and inflammatory response, resulting in elevated cytokine levels at this later stage.

Inflammation is a major pathological feature of *T. spiralis* infection, with TNFα and TGF-β playing central roles in immune regulation and fibrosis progression. TNFα, a potent pro-inflammatory cytokine, was highly upregulated in the infected control group, confirming the presence of intense inflammation. While albendazole influenced TNFα expression, *S. officinalis* extract treatment significantly downregulated TNFα. Also, the combination therapy further suppressed TNFα expression, suggesting a strong anti-inflammatory effect. These findings align with Frydas et al.‘s (2001) and Saleh et al.‘s (2024**)** studies, showing that the TNFα level was detectable on the first day p.i. and continued to increase with a maximum effect on the 37th day p.i. El Jery et al. (2020) suggested that *S. officinalis* exhibits anti-inflammatory activity through cytokine modulation.

Infected controls showed consistent TGF-β upregulation, indicative of fibrotic progression (aligning with Song et al. 2019 and in contrast with Saleh et al. 2024). Albendazole monotherapy moderately reduced TGF-β, while *S. officinalis* extract more effectively downregulated it. Combination therapy exhibited mild regulatory effects, suggesting *S. officinalis* may balance fibrotic pathways, consistent with its reported role in preventing excessive fibrosis while supporting tissue repair. This aligns with findings that *S. officinalis* reduces bleomycin-induced lung fibrosis in rats (Bahri et al. 2020), normalizes oxidative stress, and decreases collagen deposition, potentially through TGF-β signaling modulation. Furthermore, studies on *Salvia miltiorrhiza* demonstrate that its compounds inhibit TGF-β1, attenuating fibrosis in various tissues (Wu et al. 2019). Collectively, these findings suggest that *Salvia* species possess anti-fibrotic properties via TGF-β signaling modulation, supporting controlled tissue repair.

IL-10, an anti-inflammatory cytokine, was highly upregulated in the infected control group, likely to counteract TNFα-driven inflammation. Albendazole treatment significantly reduced IL-10 levels, while *S. officinalis* maintained higher levels, reinforcing its immunomodulatory properties. The combination therapy further suppressed IL-10 expression, suggesting that *S. officinalis* regulates inflammation without excessive immune suppression, which aligns with previous studies on herbal treatments and immune homeostasis (Mollazadeh et al. 2019; Trinh et al. 2020). The upregulation of IL-10 in the infected control group supports the idea that *T. spiralis* promotes a regulatory immune environment to enhance its survival by dampening host immunity, consistent with previous research of Frydas et al. (2001), Song et al. (2019) and Wang et al. (2020). Both albendazole and *S. officinalis* extract treatment reduced IL-10 expression moderately. The combination therapy significantly downregulated IL-10, suggesting it effectively reversed the parasite-induced immunosuppressive state, potentially restoring immune balance and promoting parasite clearance.

Flavonoids and terpenes likely contribute to the herb’s anti-inflammatory and antinociceptive effects (Rathee et al. 2009; Boufadi et al. 2021). Ursolic and rosmarinic acid from *S. officinalis* exhibit anti-inflammatory activity twice as potent as indomethacin (Baricevic et al. 2001). Studies in mice demonstrate that *S. officinalis* flavonoids reduce inflammation in the carrageenan model (Azevedo et al. 2013; Mansourabadi et al. 2015), and topical rosmarinic acid inhibits epidermal inflammation (Osakabe et al. 2004). Flavonoids can also inhibit cyclooxygenase and TNF secretion, further controlling inflammation (Dashti-Rahmatabadi et al. 2009).

This study suggests that *S. officinalis* possesses strong antifibrotic and anti-inflammatory properties, by downregulating FN1, TNF-α, and TGF-β while maintaining IL-10 expression, making it a valuable adjunct therapy for trichinellosis. While albendazole remains essential for parasite clearance, its limited impact on fibrosis and inflammation highlights the need for complementary treatments. The combination therapy of albendazole and *S. officinalis* demonstrated superior regulation of fibrotic and inflammatory markers, supporting its potential use in parasite-induced muscle damage and chronic infections.

## Supplementary Information

Below is the link to the electronic supplementary material.

Supplementary Material 1

## Data Availability

No datasets were generated or analysed during the current study.

## Declarations

All procedures and experimental protocols concerning this work were approved by the ASU Research Ethics Committee (ASU-REC), Egypt (Ethical Committee Permission No.: ASU-SCI/ZOOL/2023/10/3).
