# Supplementary material for: Potential antioxidant and anti-inflammatory impacts of Salvia officinalis leaves extract on mice experimentally infected with Trichinella spiralis
Source: Vet Res Commun. 2025 Sep 30;49(6):337. doi: 10.1007/s11259-025-10875-w (PMC12484373; doi:10.1007/s11259-025-10875-w)
Supplement: Supplementary file 1 — Supplementary Material 1 [file 11259_2025_10875_MOESM1_ESM.docx]

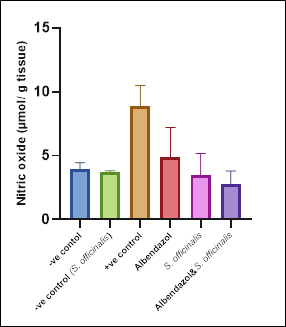

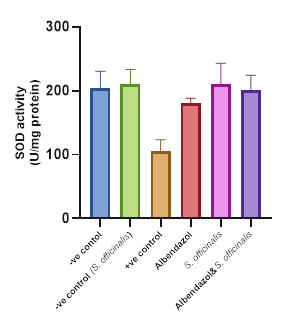

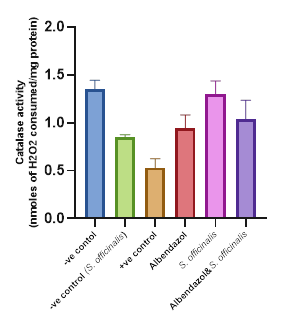

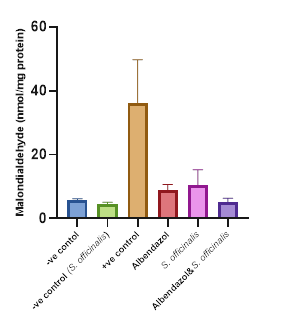

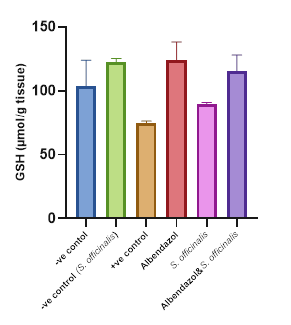


**e**

**d**

**c**

**b**

**a**

Figure 1: The effects of the different treatments on various oxidative stress markers in liver tissues at day 7 p.i. (a) superoxide dismutase (b) catalase (CAT), (c) reduced glutathione (GSH), (d) malondialdehyde (MDA), and (e) nitric oxide (NO). The data were expressed as mean ± SD.

**e**


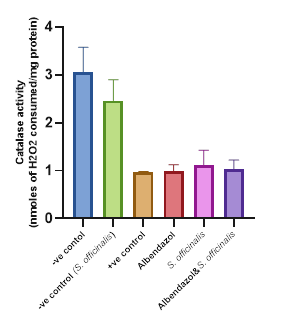

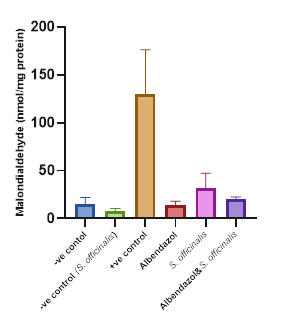

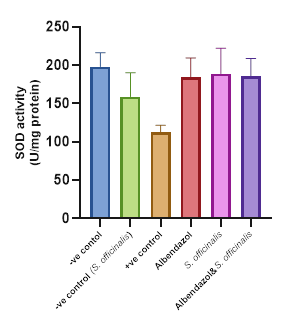

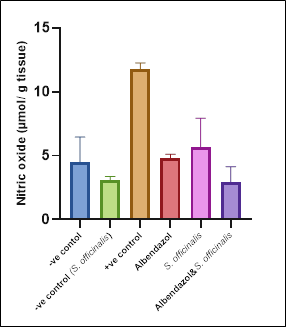

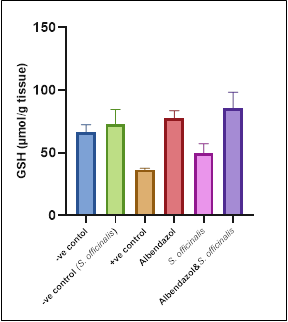


**b**

**d**

**c**

**a**

Figure 2 : The effects of the different treatments on various oxidative stress markers in liver tissues at day 37 p.i. (a) superoxide dismutase (b) catalase (CAT), (c) reduced glutathione (GSH), (d) malondialdehyde (MDA), and (e) nitric oxide (NO). The data were expressed as mean ± SD.

**A**

**B**


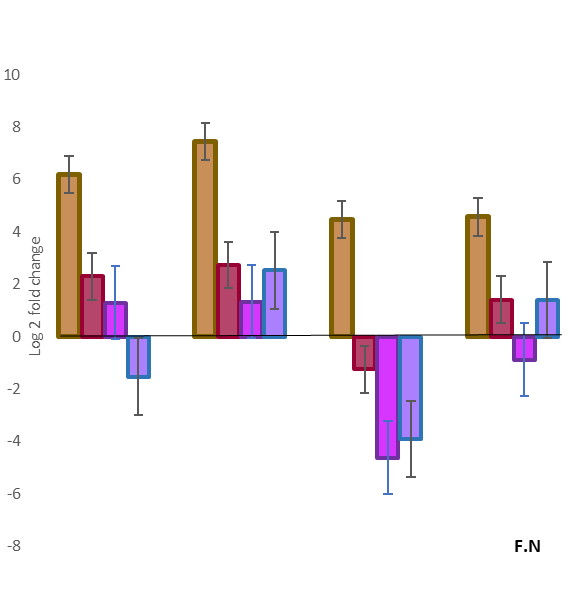

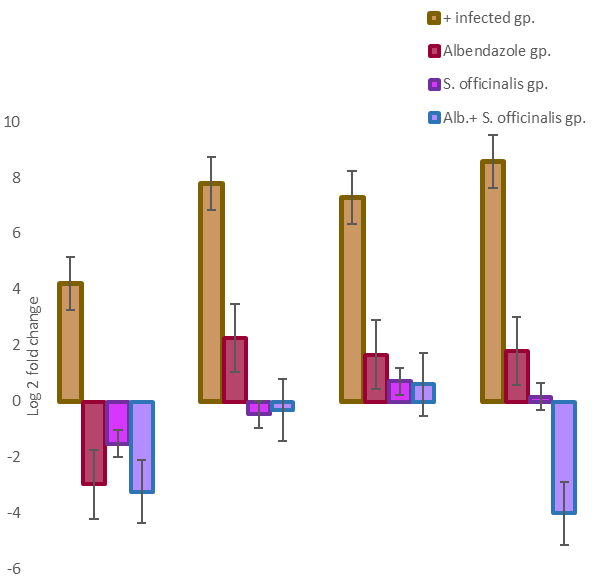


**TNFα**

**F.N**

**TGF-β**

**IL-10**

**TNFα**

**IL-10**

**TGF-β**

Figure 3: Graph showing the log2 fold change of FN1, TNF-α, TGF-β, and IL-10 (A) in intestinal tissues 7 days p.i (B) in muscle tissues 37 days p.i across experimental groups
